# Mathematical Modelling of Cerebral Blood Circulation and Cerebral Autoregulation: Towards Preventing Intracranial Hemorrhages in Preterm Newborns

**DOI:** 10.1155/2014/965275

**Published:** 2014-07-13

**Authors:** Renée Lampe, Nikolai Botkin, Varvara Turova, Tobias Blumenstein, Ana Alves-Pinto

**Affiliations:** ^1^Research Unit of Buhl-Strohmaier-Foundation for Children Orthopedics and Cerebral Palsy, Department of Orthopaedics, Clinic “Rechts der Isar”, Technische Universität München, 81675 München, Germany; ^2^Chair of Mathematical Modelling, Center for Mathematics, Technische Universität München, 85748 Garching, Germany

## Abstract

Impaired cerebral autoregulation leads to fluctuations in cerebral blood flow, which can be especially dangerous for immature brain of preterm newborns. In this paper, two mathematical models of cerebral autoregulation are discussed. The first one is an enhancement of a vascular model proposed by Piechnik et al. We extend this model by adding a polynomial dependence of the vascular radius on the arterial blood pressure and adjusting the polynomial coefficients to experimental data to gain the autoregulation behavior. Moreover, the inclusion of a Preisach hysteresis operator, simulating a hysteretic dependence of the cerebral blood flow on the arterial pressure, is tested. The second model couples the blood vessel system model by Piechnik et al. with an ordinary differential equation model of cerebral autoregulation by Ursino and Lodi. An optimal control setting is proposed for a simplified variant of this coupled model. The objective of the control is the maintenance of the autoregulatory function for a wider range of the arterial pressure. The control can be interpreted as the effect of a medicament changing the cerebral blood flow by, for example, dilation of blood vessels. Advanced numerical methods developed by the authors are applied for the numerical treatment of the control problem.

## 1. Introduction

Cerebral flow autoregulation is a process which aims to maintain proper and stable cerebral blood flow. By means of cerebral flow autoregulation, the body is able to deliver sufficient blood containing oxygen and nutrients to the brain tissue for metabolic needs and remove CO_2_ and other waste products. The most important objective of autoregulation is maintaining an appropriate level of brain perfusion, which is essential for life, since the brain has a high metabolic demand. It should be noticed that the brain is very sensitive to over- and underperfusion.

In recent years, mathematical modeling of autoregulation of cerebral blood flow (CBF) becomes very popular. Various methods like, for example, time- and frequency-domain analysis (see [1]) and principal dynamic modes (see [2]) are applied to analyze the dynamic relationship between the arterial blood pressure and cerebral blood flow velocity. Also, the so-called lumped parameter models based on the analogy to electric circuits (see, e.g., [3–5]) are widely used to simulate cerebral flow autoregulation mechanisms. Usually, the models are based on nonlinear ordinary differential equations (ODE) describing the flow balance in different parts of the cerebral vascular system (see, e.g., [6]). Autoregulation effects arise due to feedback relations governing the vascular volume (*V*). It is supposed that *V* is proportional to the square of mean vascular radius (*r*), and the resistance (*R*) is inversely proportional to the fourth power of *r* according to the Hagen-Poiseuille law. Therefore, *R* ~ 1/*V* ^2^, which yields the relation CBF ~ *V* ^2^, or *V* ~ CBF^0.5^. It should be noticed that the experimental value of the exponent in the last relation is about 0.38 (see [7]). This difference points out to the oversimplification of the cerebrovascular bed considered as the parallel arrangement of several equal microvessels. A more anatomically plausible, hierarchical model of the cerebrovascular bed is proposed in [7]. It consists of 19 compartments representing the whole range of vascular sizes and respective CO_2_ reactivities derived from literature data.

In this paper, we use the model from [7] in the context of autoregulation. We consider two methods for describing the dependence of the vascular radius on the arterial blood pressure. First, a polynomial dependence supported by fitting the corresponding coefficients to experimental data on CBF autoregulation in newborns is used. Introducing a hysteretic behavior into the resulting model is tested. Second, the ODE model from [6] is coupled with the model of [7]. A conflict control problem for such a model is considered. The control is interpreted as intake of a drug controlling the vascular volume, whereas unpredictable changes in arterial pressure are considered as disturbance.

Thus, the paper presented can be considered as a research demonstrating the idea to couple phenomenological compartment ODE models with physically consistent blood flow models derived from hydrodynamic laws. This opens the following perspectives. We are going to adapt the hydrodynamic part of the model to various types of cerebrovascular beds including germinal matrices of premature infants. This should be possible due to the flexibility of hydrodynamic models that can use precise descriptions of blood vessel networks, account for the assistance of vessel muscles in the blood circulation, or, conversely, assume muscular passivity of blood vessels. Moreover, different non-Newtonian and micropolar fluids can be used. The most important feature of hydrodynamic models is that they give the exact value of the blood pressure in all vessel types. Therefore, it becomes possible, based on the information about vessel sizes, to find the highest stress concentration in vessel walls. Then, using methods of elasticity and strength theory, breaking of the most fragile vessels can be predicted. On the other hand, control parameters included into the coupled model may be used to provide a regime that excludes damaging stresses in the most vulnerable vessels. Another peculiarity of the hydrodynamic part of the model is that only physical relationships between variables are imposed. Some artificial relations are not necessary. As for the phenomenological part of the model, related to ordinary differential equations, it should be refined, and phenomenological assumptions should be replaced by biophysical ones.

## 2. The Hierarchical Cerebrovascular Model

In this section, a hierarchical cerebrovascular model proposed in [7] is outlined. The derivation of expressions defining the CBF and the pressure in each compartment is explained. Notice that such a derivation is omitted in [7].

**Figure 1:**
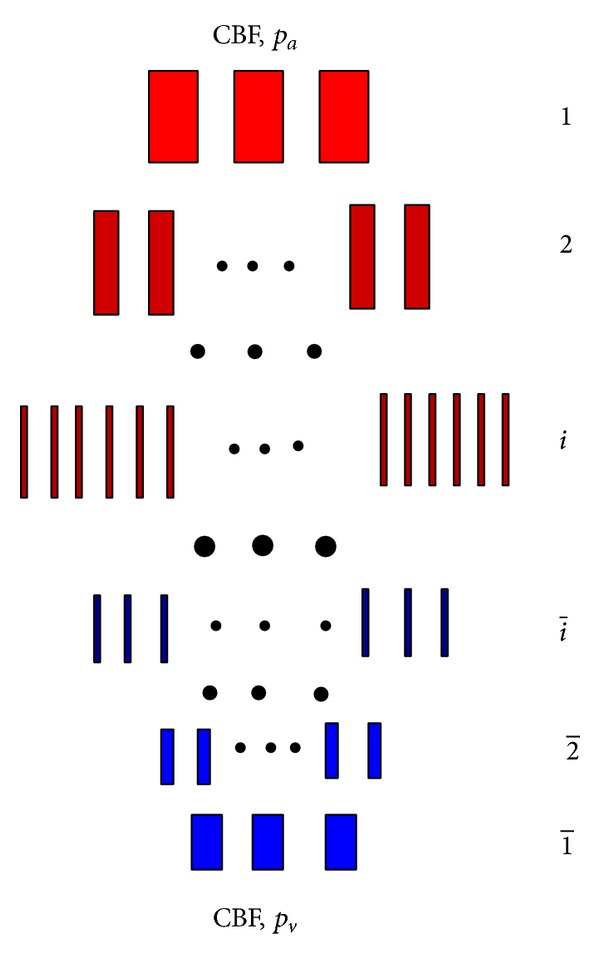
Cerebrovascular bed considered in [7]. The arterial levels are shown in red and the venous ones are depicted in blue. Notice that the venous levels are numbered from bottom to top. The blood inflow on the top equals the blood outflow on the bottom. The arterial and venous pressures *p*_*a*_ and *p*_*v*_ are applied on the top and bottom, respectively.

Let *N* ($\bar{N}$) be the number of arterial (venous) levels (see Figure 1), and $M=N+\bar{N}$. Denote by *p*_*i*_ the pressure at the input of the *i*th level. Notice that either *i* ∈ {1,2,…, *N*} (arteries) or $i\in \{\bar{1},\bar{2},\ldots ,\bar{N}\}$ (veins). Assume that *p*_1_ = *p*_*a*_ and $p_{\;\bar{1}}=p_{v}$, where *p*_*a*_ and *p*_*v*_ are the arterial and venous pressures, respectively. Let *R*_*i*_, *m*_*i*_, *r*_*i*_^0^, *c*_*i*_, and *l*_*i*_ be the resistance, the number, the radius, the CO_2_ reactivity, and the length of vessels of the *i*th level, respectively. The radius, *r*_*i*_, of vessels of the* i*th level depends on the partial CO_2_ pressure, *P*_CO_2__, and is computed by the formula

(1) $$\begin{array}{c} r_{i}=r_{i}\left(P_{\text{C}{\text{O}}_{2}}\right)=r_{i}^{0}\cdot \left(1+c_{i}\cdot P_{\text{C}{\text{O}}_{2}}\right). \end{array}$$

According to the Poiseuille law, the resistance of vessels is given by the formula *R*_*i*_ = 8*μl*_*i*_/*πr*_*i*_^4^, where *μ* is the dynamic viscosity of blood.

If CBF is known, the following relations obviously hold (Kirchhoff's law):

(2) $$\begin{array}{c} p_{n+1}=p_{1}-\text{CBF}\overset{n}{\underset{i=1}{\sum }}\frac{R_{i}}{m_{i}},\;\;p_{\;\bar{n+1}}=p_{\;\bar{1}}+\text{CBF}\overset{\bar{n}}{\underset{i=\bar{1}}{\sum }}\frac{R_{i}}{m_{i}}. \end{array}$$

Therefore, CBF can be found from the following equality of pressures on the arteriovenous junction:

(3) $$\begin{array}{c} p_{1}-\text{CBF}\overset{N}{\underset{i=1}{\sum }}\frac{R_{i}}{m_{i}}=p_{\;\bar{1}}+\text{CBF}\overset{\bar{N}}{\underset{i=\bar{1}}{\sum }}\frac{R_{i}}{m_{i}}. \end{array}$$

Therefore,

(4) $$\begin{array}{c} \text{CBF}=\left(p_{1}-p_{\;\bar{1}}\right){\left(\overset{N}{\underset{i=1}{\sum }}\frac{R_{i}}{m_{i}}+\overset{\bar{N}}{\underset{i=\bar{1}}{\sum }}\frac{R_{i}}{m_{i}}\right)}^{-1}. \end{array}$$

Finally, if we number all levels from the top down and remember that *R*_*i*_ = 8*μl*_*i*_/*πr*_*i*_^4^, we have

(5) CBF=(pa−pv)(∑i=1M8μliπmiri4)−1,

(6) pn+1=pa−CBF∑i=1n8μliπmiri4, n=0,…,M.

Notice that, formally, *p*_1_ = *p*_*a*_ and *p*_*M*+1_ = *p*_*v*_.

Combining formulae (1), (5), and (6) yields

(7) pn+1(pa,pv,PCO2)=pa−(pa−pv) ×(∑i=1M8μliπmiri4)−1∑i=1n8μliπmiri4,n=0,…,M,

where *r*_*i*_ are computed by formula (1).

**Figure 2:**
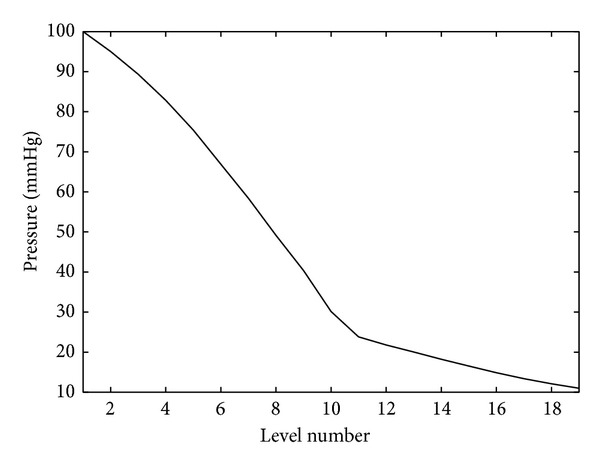
Pressure on the arterial (*n* = 1,…, 10) and venous (*n* = 11,…, 19) levels.

Figure 2 shows the blood pressure depending on the vascular level. The computation is performed using formula (7). Here, we set *p*_*a*_ = 100  [mmHg]  , *p*_*v*_ = 10  [mmHg]  , and *P*_CO_2__ = 0. The values of *l*_*i*_, *m*_*i*_, *r*_*i*_^0^, and *c*_*i*_, *i* = 1,…, *M* = 19, are taken from [7].

**Figure 3:**
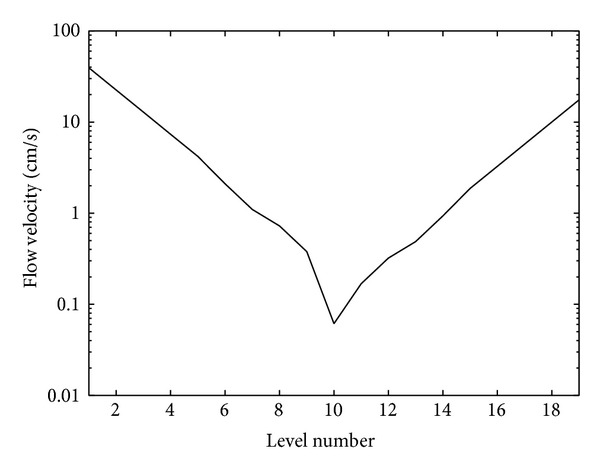
Flow velocity depending on the level.

Figure 3 presents the flow velocity in each vessel depending on the level. The velocity is computed as CBF/(*m*_*i*_ *πr*_*i*_^2^), where CBF is given by formula (5). The condition *P*_CO_2__ = 0 is assumed.

**Figure 4:**
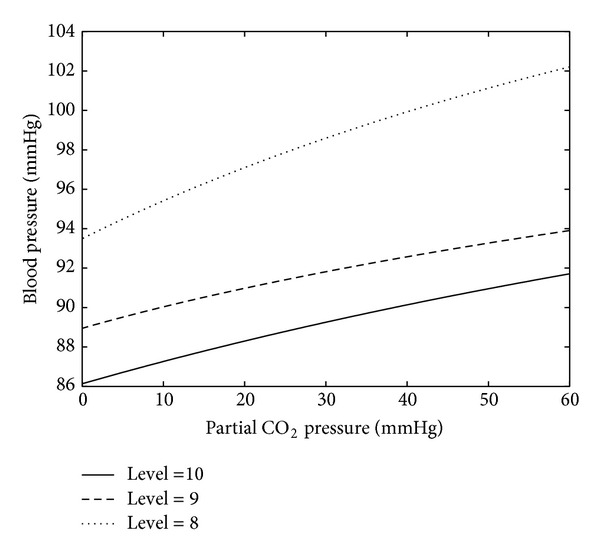
Blood pressure on levels 8, 9, and 10 depending on the partial CO_2_ pressure (*P*_CO_2__).

Figure 4 shows the dependence of the pressure in the 8th, 9th, and 10th compartments on the partial CO_2_ pressure. The computation is performed using formula (7).

**Figure 5:**
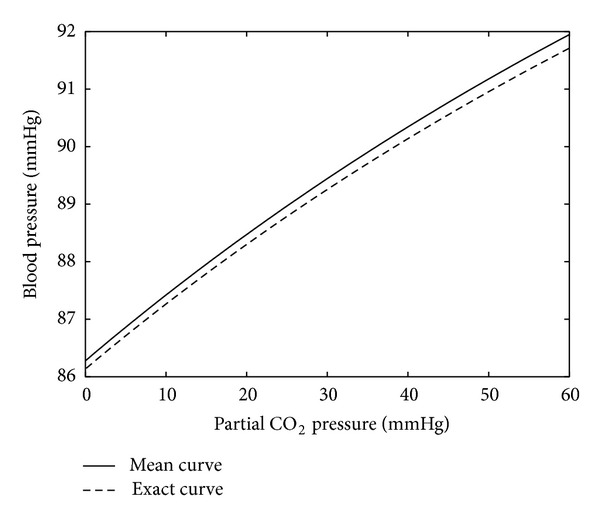
Blood pressure on the 10th level versus the partial CO_2_ pressure. The solid curve is obtained by averaging of test runs corresponding to random variations in the model data by 20%. The dashed curve corresponds to the reference values of the parameters.

Figure 5 shows some statistical stability of the model with respect to the variation of the parameters *m*_*i*_, *l*_*i*_, *r*_*i*_^0^, and *c*_*i*_. These parameters were randomly varied around the reference values reported in [7]. The uniform distribution of the variations was used, and the amplitude of the variations was equal to 20%. The dependence of the pressure on the 10th level on the partial CO_2_ pressure was computed in each test, and 200 random tests were done. The solid curve presents the average of the curves computed in each test. The dashed curve corresponds to the reference values of the parameters. The computations are done using formula (7).

## 3. Autoregulation Using a Polynomial Feedback

Now, the model described in the previous section is extended by adding a polynomial dependence of the vascular radiuses on the arterial blood pressure and adjusting the polynomial coefficients to experimental data to gain the autoregulation behavior. Additionally, a Preisach hysteresis operator is introduced into the resulting model to simulate the hysteretic dependence of the cerebral blood flow on the arterial pressure. Throughout this section, the assumption *P*_CO_2__ = 0 holds.

Let *p*_a_* be a reference value of the arterial pressure and *r*_i_*, *i* = 1,…, *M*, the vascular radiuses corresponding to *p*_a_*. Assume that the dependence of *r*_*i*_ on *p*_*a*_ is chosen as follows:

(8) $$\begin{array}{c} r_{i}=r_{i}^{*}{\left[1+\lambda \left(p_{a}-p_{a}^{*}\right)\right]}^{-1/4}, \end{array}$$

where *λ* is a parameter. Such a form of *r*_*i*_ is motivated by the fact that they appear as fourth powers in the expression (5) so that the CBF becomes proportional to (*p*_*a*_ − *p*_*v*_)/(1 + *λ*(*p*_*a*_ − *p*_a_*)), and therefore the choice *λ* = 1/(*p*_a_* − *p*_*v*_) stabilizes CBF. Formally, this choice of *λ* yields the relation

(9) $$\begin{array}{c} r_{i}=r_{i}^{*}{\left[\frac{\left(p_{a}^{*}-p_{v}\right)}{\left(p_{a}-p_{v}\right)}\right]}^{1/4}, \end{array}$$

which implies (for all *p*_*a*_) the equality

(10) $$\begin{array}{c} \text{CBF}\left(p_{a}\right)\equiv \text{CB}{\text{F}}^{*}≔\left(p_{a}^{*}-p_{v}\right){\left(\overset{M}{\underset{i=1}{\sum }}\frac{8\mu l_{i}}{\pi m_{i}r_{i}^{*\;4}}\right)}^{-1}. \end{array}$$

To adapt the model to experimental data (see [8–10]), consider the following modification:

(11) ri=ri∗[(pa∗−pv)(pa−pv)]1/4 ×[1+a1(pa−pa∗)+a2(pa−pa∗)2+a3(pa−pa∗)3].

Therefore,

(12) $$\begin{array}{c} \text{CBF}\left(p_{a},a_{1},a_{2},a_{3}\right)=\left(p_{a}-p_{v}\right)k_{\text{age}}{\left(\overset{M}{\underset{i=1}{\sum }}\frac{8\mu l_{i}}{\pi m_{i}r_{i}^{4}}\right)}^{-1}, \end{array}$$

where *r*_*i*_ = *r*_*i*_(*p*_*a*_, *a*_1_, *a*_2_, *a*_3_) is given by formula (11) and *k*_age_ is a scale factor to adjust CBF to the age of infants. Set *p*_a_* = 34  [mmHg]  , *p*_*v*_ = 5  [mmHg]  , and *k*_age_ = 0.08, which corresponds to hemodynamic system of premature infants of 31–34 weeks' gestational age with 260 g brain weight and CBF of 15.5 mL/100 g/min (cf. [9, 10]). The values of *l*_*i*_, *m*_*i*_, and *r*_i_*, *i* = 1,…, *M* = 19, are taken from [7]. The coefficients *a*_1_, *a*_2_, and *a*_3_ are fitted through the minimization of the residual

(13) $$\begin{array}{c} R\left(a_{1},a_{2},a_{3}\right)=\overset{5}{\underset{k=1}{\sum }}{\left[\text{CBF}\left(p_{a}^{k},a_{1},a_{2},a_{3}\right)-\text{CB}{\text{F}}^{k}\right]}^{2}, \end{array}$$

where the pairs (*p*_*a*_^*k*^  [mmHg]  , CBF^*k*^  [mL/s]  ), *k* = 1,…, 5, are chosen according to [9] as follows:

(14) $$\begin{array}{c} \left(20,0.24\right);\left(30,0.67\right);\left(34,0.67\right);\left(38,0.67\right);\left(50,2\right). \end{array}$$

The values (in [1/Pa]) of the minimizers of (13) read

(15) a1=  8.38387799566571010e−7,a2=  5.71754835857535438e−9,a3=  3.51842656454501967e−11.

It should be noticed that the polynomial appearing in square brackets in (11) with coefficients (15) represents a sigmoidal function providing autoregulation. Thus, the polynomial ansatz is a method of constructing an appropriate sigmoidal function.

**Figure 6:**
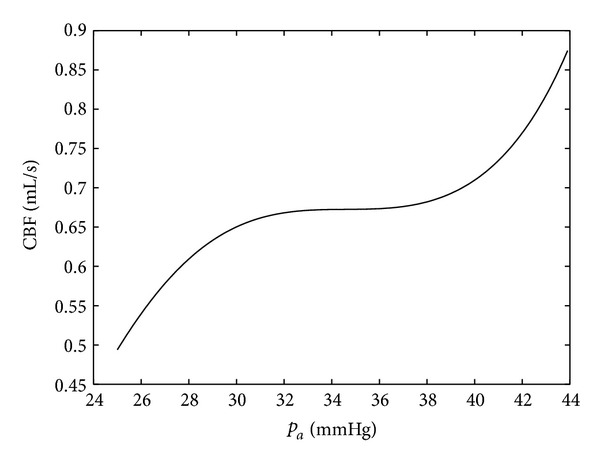
Simulation of the effect of autoregulation when using the feedback law (11).

Figure 6 shows the effect of autoregulation when using the feedback law given by formulae (11) and (12) with the coefficients (15).

**Figure 7:**
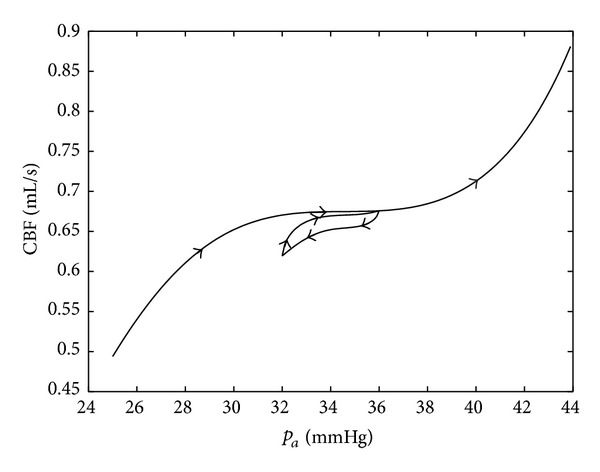
Autoregulation with hysteresis.

Figure 7 shows the same simulation as in Figure 6, but a hysteretic behavior of the process is added by applying a Preisach hysteresis operator. Thus, if the arterial pressure increases, the output follows the main path (cf. Figure 6). If the pressure starts to go down at some point, the output path goes back below the main path. If the pressure again increases, the output returns to the main path and follows it. Notice that Preisach hysteretic relations express wetting/drainage processes in structures containing a large number of small vessels and capillaries. A fast numerical method implementing the Preisach hysteresis operator is developed in [11]. The simulation of hysteretic behavior was motivated by experimental results reported in papers [12–14].

In [12], an asymmetry of autoregulation during spontaneous increases and decreases of cerebral perfusion pressure (CPP) in brain injury patients was experimentally shown. The autoregulatory response was significantly greater during increase than during decrease in CPP.

Experimental data of [13] based on a pharmacological approach to evaluate dynamic cerebral autoregulation (dCA) gain to transient hypotension and hypertension in healthy patients showed that cerebral autoregulation is different for rising and falling blood pressure. Namely, dCA gain to transient hypotension was consistently greater than dCA gain to transient hypertension.

According to [14], a strongly asymmetric dynamic response of the cerebral autoregulation was seen in the majority of patients with head injury. It might also have been present, albeit to a lesser degree, in the normal subjects. The findings suggest that nonlinear effects may be present in the operation of the cerebral autoregulation mechanism.

## 4. Autoregulation Using an ODE Model by Ursino and Lodi

In this section, the ODE model from [6] is coupled with the model of [7] (cf.  Section 2). The aim of this section is to compare simulation results produced by our coupled model with the results of [6]. Thus, the data reported in [6] are used in our coupled model. These data correspond to adult persons, and therefore the autoregulation area approximately extends from 50 to 140 [mmHg], and CBF is around 11.4 [mL/s].

The variables and parameters used in [6] are the following:

 
*P*_*a*_: the arterial pressure (input),
 
*P*_vs_: the dural sinus pressure (parameter) = 6.0 mmHg,
 
*P*_CO_2__: the partial pressure of CO_2_ (parameter) = 0, 10, 20, 30, and 40 mmHg,
 
*V*_*a*_: arterial-arteriolar volume (variable),
 
*P*_ic_: the intracranial pressure (variable),
 
*P*_*c*_: capillary pressure (variable),
 
*C*_*a*_: the arterial compliance (variable),
 
*R*_*a*_: the arterial resistance (variable),
 
*q*: the cerebral blood flow (CBF) (variable),
 
*x*: the CBF deviation normalized to the basal level (variable),
 
*σ*(*x*): a sigmoidal static function,
 Δ*C*_*a*_: the amplitude of the sigmoidal curve, see (24),
 
*k*_*σ*_: the central slope = Δ*C*_*a*_/4, see (24),
 
*G*: the maximum autoregulation gain = 1.5 mL/mmHg,
 Δ*C*_*a*1_: 0.75 mL/mmHg,
 Δ*C*_*a*2_: 0.075 mL/mmHg,
 
*C*_*an*_: the central value of the sigmoidal curve = 0.15 mL/mmHg,
 
*R*_*f*_: the resistance to the formation of cerebrospinal fluid = 2.38 · 10^3^ mmHg*·*s/mL,
 
*R*_pv_: the proximal venous resistance = 1.24 mmHg*·*s/mL,
 
*R*_*o*_: the resistance to the outflow of cerebrospinal fluid = 526.3 mmHg*·*s/mL,
 
*I*_*i*_: the injection rate of cerebrospinal fluid = 1/30 mL/s,
 
*q*_*n*_: the value of CBF required by tissue metabolism = 12.5 mL/s,
 
*τ*: the time constant of the regulation = 20 s,
 
*α*: a fitting constant = 0.2,
 
*k*_*E*_: 0.11 mL^−1^.

The new model consists of the same ODEs as the model from [6],

(16) dCadt=1τ[σ(x)−Ca],

(17) dPicdtkEPic1+CakEPic ×[CadPadt+dCadt(Pa−Pic)+Pc−PicRf−Pic−PvsRo+Ii],

and the relations

(18) x=q−qnqn,

(19) q=CBF(Pa,αVa),

(20) Va=Ca·(Pa−Pic),

(21) Pc=PaRpv+PicRaRpv+Ra,

(22) Ra=RA(αVa).

Here, the relations (19) and (22) replace oversimplified formulas for *q* and *R*_*a*_ from [6]. The definitions of the functions CBF (returns the cerebral blood flow) and *RA* (returns the vascular resistance) are taken from Section 2. They are given by the formulas

(23) CBF(Pa,λ)=(Pa−Pc)RA(λ),RA(λ)=∑i=1M8μliπmi[λ·ri0·(1+ci·PCO2)]4,

where *m*_*i*_, *l*_*i*_, *r*_*i*_^0^, and *c*_*i*_ are the number, the length, the radius, and the CO_2_ reactivities of vessels of the *i*th level, respectively (see [7]).

The function *σ* is defined by the relations

(24) σ(x)=(Can+ΔCa/2)+(Can−ΔCa/2)exp⁡(G·x/kσ)1+exp⁡(G·x/kσ),ΔCa={ΔCa1if  x≤0,ΔCa2if  x>0,kσ={ΔCa14if  x≤0,ΔCa24if  x>0.

The initial state is *C*_*a*_(0) = 0.15 mL/mmHg and *P*_ic_(0) = 9.5 mmHg.

Thus, the difference with the model developed in [6] consists in the computation of the cerebral blood flow *q* and the arterial resistance *R*_*a*_ using the model of cerebrovascular bed considered in [7]. In contrast to [6], *q* and *R*_*a*_ are defined by the relations (19) and (22), respectively. Additionally, it was assumed that the vascular radiuses are proportional to the square root of the arterial-arteriolar volume; that is, $\lambda =\alpha \sqrt{V_{a}}$ in (23).

**Figure 8:**
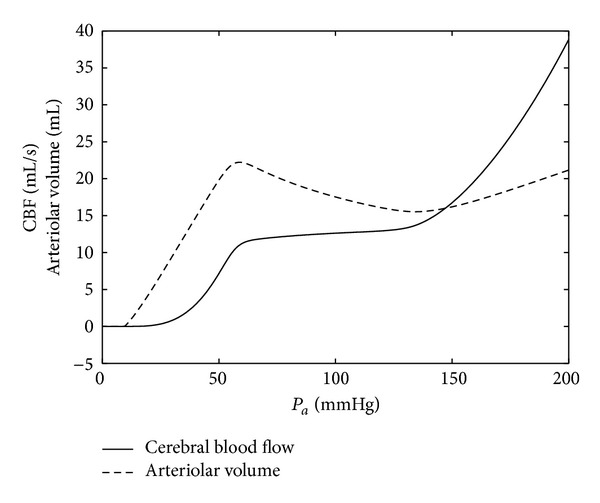
Autoregulation using the new ODE model. The slope of *P*_*a*_(*t*) is equal to 2/3 mmHg/s, and *P*_CO_2__ = 0.

Figure 8 shows two outputs of the model (16) and (17) versus *P*_*a*_. The input signal *P*_*a*_(*t*) is a linear function with the slope equal to 2/3 mmHg/s, and the partial pressure of CO_2_ equals zero.

**Figure 9:**
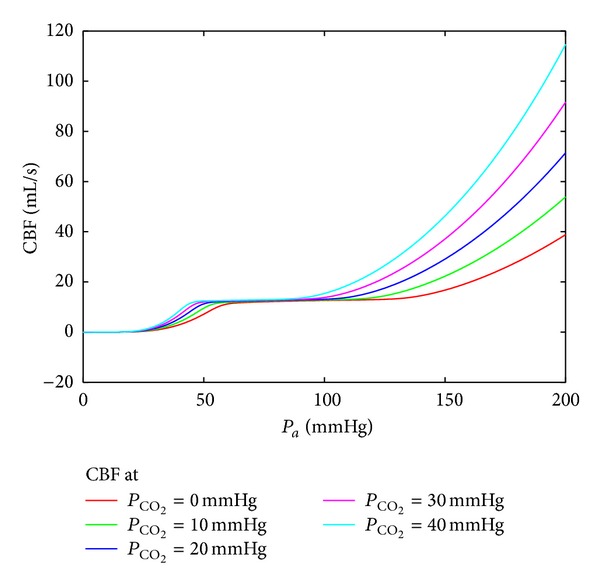
Autoregulation using the ODE model. The input function *P*_*a*_(*t*) is the same as in the previous simulation, and *P*_CO_2__ assumes the values 0, 10, 20, 30, and 40 [mmHg].

Figure 9 presents five outputs of the model (16) and (17) corresponding to the following magnitudes of the partial CO_2_ pressure: 0, 10, 20, 30, and 40 [mmHg]. The input signal *P*_*a*_(*t*) is the same as in the previous simulations.

## 5. Conflict Control Setting

The crucial variable defining the behavior of the system (16)–(22) is the arterial-arteriolar volume *V*_*a*_ controlled by the arterial compliance *C*_*a*_; see (20). The behavior of *C*_*a*_ can be influenced by a control parameter, *u*, added to the right-hand-side of (16). The effect of *u* can be interpreted as the intake of a drug increasing/decreasing the response of the CBF autoregulation system to the deviation of *P*_*a*_. Moreover, assume now that the realization of *P*_*a*_ is formed by an interfering factor that intends to crash the nominal CBF. Assume that this factor “chooses” the rate, *v*, of *P*_*a*_ at each time instant. Additionally, observe that simulations of the model (16)–(22) show small variations of the variable *P*_*ic*_, and therefore (17) can be preliminary neglected. Summarizing these assumptions, arrive at the following conflict control system:

(25) dCadt=1τ[σ(x(Pa,Ca))−Ca]+u,dPadt=v,

where the function *x*(*P*_*a*_, *C*_*a*_) is defined by formulae (18), (19), and (20) with *P*_ic_ = 6 mmHg. We assume that the control, *u*, and the disturbance, *v*, are restricted as follows: |*u*(*t*)|≤0.01 and |*v*(*t*)|≤0.6. The objective of *u* is the minimization of the functional

(26) $$\begin{array}{c} J=\underset{t\in [0,t_{f}]}{\max}\left\{\left|q\left(t\right)-q_{n}\right|\cdot \Psi \left(P_{a}\left(t\right)\right)\right\}, \end{array}$$

whereas *v* strives to maximize it. Here, *t*_*f*_ is the termination time of the control process, and Ψ is a cutoff function such that Ψ(*s*) = 1 if *s* ∈ [60,180] and it rapidly falls to zero outside of the above interval. Notice that the usage of Ψ means that the deviation |*q*(*t*) − *q*_*n*_| is not assumed to be small outside of the reasonable range of the arterial pressure.

The system (25) contains nonlinear dependencies given by a computer subroutine (cf. the computation of *q*). Moreover, a disturbance is involved into the dynamics. Thus, the application of traditional control design methods based on Pontryagin's maximum principle is not applicable. Nevertheless, the dynamical programming principle related to Hamilton-Jacobi-Bellman-Isaacs (HJBI) equations is suitable in this case. The application of this technique requires stable grid methods for solving HJBI equations arising from conflict control problems. Such methods are developed by the authors (see [15, 16]) and can be applied to the problem (25)-(26). The next section outlines an upwind grid method for the solution of HJBI equations.

### 5.1. Finite-Difference Scheme

Notice that the state vector of the problem (25)-(26) is two-dimensional. For generality, consider *n*-dimensional conflict control problem with the dynamics

(27) x˙=f(t,x,u,v), t∈[0,tf],  x∈Rn,u∈A⊂Rμ,  v∈B⊂Rν,

and the gain defined as follows:

(28) $$\begin{array}{c} c\left(t,x\right)=\underset{A}{\min\;}\underset{B}{\max}\underset{\tau \in \left[t,t_{f}\right]}{\max}\theta \left(\tau ,x\left(\tau \right)\right). \end{array}$$

Here, *u* and *v* are the control parameters of the minimizing and maximizing players, respectively, *A* and *B* are feedback strategies of the players, and (*t*, *x*) is the start position of the control process.

Let *ρ* and *h*≔(*h*_1_,…, *h*_*n*_) be time and space discretization step sizes. Let *C* ^*h*^ be a grid function. Define an upwind operator, *F*, as follows:

(29) F(Ch;t,ρ,h1,…,hn)(x)=Ch(x) +ρ max⁡v∈B min⁡u∈A∑k=1n(pkRfk++pkLfk−),

where

(30) a+=max⁡{a,0},  a−=min⁡{a,0},pkR=[Ch(x1,…,xk+hk,…,xn)−Ch(x1,…,xk,…,xn)]hk,pkL=[Ch(x1,…,xk,…,xn)−Ch(x1,…,xk−hk,…,xn)]hk,

and *x* runs over all grid points.

Let *M* = *t*_*f*_/*ρ* + 1. Denote *t*_*m*_ = *mρ*,  *m* = 0,…, *M*, and introduce the following notation:

(31) cm(xj1,…,xjn)=c(tm,j1h1,…,jnhn),θm(xj1,…,xjn)=θ(tm,j1h1,…,jnhn);

that is, *x*_*j*_1__,…, *x*_*j*_*n*__ runs over all grid points. The numerical scheme is the following:

(32) $$\begin{array}{c} c^{m-1}=\max\left\{F\left(c^{m};t_{m},\rho ,h_{1},\ldots ,h_{n}\right),\theta^{m}\right\},\;\;c^{M}=\theta^{M}. \end{array}$$

The proof of the convergence of *c* ^*m*^ to the gain *c* is given in [15, 16]. Optimal feedback strategies of the players can be computed by storing a minimizer *u* and a maximizer *v* in (29) at each sampled time instant and each grid point. Another way of the construction of optimal feedback strategies is the so-called extremal aiming procedure (see, e.g., [16] for an explanation).

### 5.2. Result

**Figure 10:**
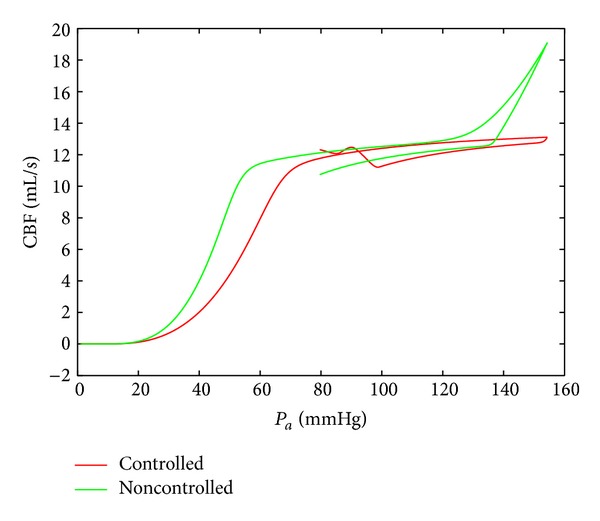
The comparison of the behavior of the controlled and uncontrolled CBF autoregulation systems.

Figure 10 shows the result of the application of the above sketched technique to the minimization of the functional (26). The input signal *P*_*a*_(*t*) grew from zero to 150 [mmHg] with a constant rate and then fell to 80 [mmHg]. One can observe the extension of the horizontal plateau in the case of applying the control. Thus, the computed feedback control, *u*(*C*_*a*_, *P*_*a*_), where *P*_*a*_ is measured and *C*_*a*_ is defined from the model (25), allows us to fit the intake of the drug to pressure jumps.

## 6. Conclusions

Simple models based on Kirchhoff's law and Hagen-Poiseuille flow of Newtonian fluids are studied here in the context of CBF autoregulation. Such models do not require a great amount of data. Information on the approximate number of vessels, their size, length, and reactivity is already available for cerebral vascular system including germinal matrices of preterm newborns. Moreover, the model behavior is stable with respect to the variation of these data. Using such models allows us to estimate pressures in the germinal matrix and their dependency on the partial CO_2_ pressure. Coupling this model with models of CBF autoregulation may be useful to gain a better understanding of mechanisms of violation of hemodynamics in germinal matrices.

The future work will be related to the enhancement of the above described coupled model. First, a method for generation of hierarchical cerebrovascular networks will be developed. Then, the hydrodynamic part of the model will be extended by accounting for non-Newtonian and micropolar properties of blood (modification of Poiseuille's law). Expansion/contraction travelling waves propagating along vessels and the curvature of vessels will be included in the model. Such an enhanced model should predict the precise pressure distribution over the vessel network. The information about the length, radius, and wall thickness of vessels will allow us to find vessels having maximal stress values in their walls. These results will be used in finite element simulations to estimate the damage risk of such vessels. The optimal control setting will be enhanced in such a way that the objective will consist in keeping stresses in a safe range over all vessels.
